# The Enlistment of Insured Persons

**Published:** 1915-05-22

**Authors:** 


### LONDON PANEL COMMITTEE.

### The Enlistment of Insured Persons.

The perennial difficulty of the proper basis of deduction from panel practitioners' lists in respect of an unknown number of insured persons who have joined the Forces again occupied the attention of the London Panel Committee at its meeting on May 18. The Panel Committee having declined to concur in the proposal of the Insurance Committee to reduce the quarterly advances to 1s. instead of 1s. 9d., the latter body suggested a conference, and it was now reported that this had taken place. As a result the Insurance Committee offered to make an advance of 1s. 4d. instead of 1s. The General Purposes Sub-Committee advised the Panel Committee to accept this, but at the same time to point out to the Insurance Committee:

1. The great inconvenience likely to be caused to practitioners at this time of stress by a diminution in the normal rate of advances.
2. The special injustice caused by a uniform reduction to cover the general incidence of recruiting in the case of practitioners whose lists contained a very small number of men of military age.
3. The conviction of the Panel Committee, from its knowledge of the condition of the London medical registers, that greater injustice was being caused than would be necessary if the registration of insured persons were reasonably up to date and accurate.
4. The desirability of doctors' lists being speedily corrected so that, at least during the last quarter of the year, many necessary deductions might be made in respect of insured persons definitely removed by name from individual lists.

Although several members stated that, so far as their own lists were concerned, the register of the Insurance Committee did not appear to be in the hopeless state generally suggested, the Committee decided to write to the Insurance Committee in the sense indicated.

### Disciplinary Measures in the Panel Service.

It was reported that a practitioner who had been censured by the Medical Service Sub-Committee of the Insurance Committee had appealed to the Insurance Commissioners, who stated that the conclusion reached by the Sub-Committee was not established by the evidence, that the practitioner was entitled to the benefit of the doubt, and that the decision of the Insurance Committee must be reversed.

In the course of a discussion several members suggested that, if more practitioners appealed, many decisions of the Medical Service Sub-Committee would be reversed, also that the medical members of the Sub-Committee, so far from defending those of their colleagues who were brought before the tribunal, were the most severe in criticism. The representatives were asked to present a full report of their action on the Medical Service Sub-Committee in order that the Panel Committee might have an opportunity of considering the whole question.

The reply of the medical representatives, Dr. R. V. Donnellan and Dr. Ethel Bentham, however, appeared to satisfy the Committee, for they both received a sympathetic ovation on resuming their seats. They declared that their duties were often painful, and that occasionally it was not a case of defending a doctor but of finding anything to say in mitigation of his offence. Both expressed the hope that the Panel Committee would not expect them to carry out the difficult and arduous duties indefinitely, but that all the members of the Panel Committee in turn would serve on the Medical Service Sub-Committee. Only personal contact with the work would bring home a realisation of the real state of affairs.

